# Dual resonant transmissive circular dichroism in a rotationally transformed chiral metasurface

**DOI:** 10.1038/s44459-026-00101-7

**Published:** 2026-11-02

**Authors:** Muhammad Noman, Jalil Kazim, Adnan Zahid, Hassan T. Chattha, Farooq A. Tahir, Muhammad Imran, Qammer H. Abbasi

**Affiliations:** 1 James Watt School of Engineering, University of Glasgow, Glasgow, UK; 2 School of Engineering and Physical Sciences, Heriot-Watt University, Edinburgh, UK; 3 Microlynx Systems Ltd, Calgary, AB Canada; 4 School of Electrical Engineering and Computer Science, National University of Sciences and Technology, Islamabad, Pakistan

**Keywords:** Optics and photonics, Physics

## Abstract

An electrically thin bilayer chiral metasurface is presented for dual resonant, same handedness circular-polarization-selective transmission in the X-band. The 11 × 11 mm^2^ connected unequal-arm unit cell produces forward right-handed circularly polarized (RHCP) circular dichroism (CD) maxima of 0.72 and 0.75 at 9.672 and 10.636 GHz within a common transmission window, with reciprocal backward left-handed circularly polarized (LHCP) selectivity and a complete CD null at 10.3 GHz. A controlled four-configuration comparison shows that the compound front–back transformation establishes chiral interlayer coupling, whereas unequal terminal paths enable the second modal contribution. Surface-current distributions identify a lower even mode, an intermediate mixed state, and an upper odd interlayer-coupled mode. A geometry-derived equivalent-circuit model reproduces the broadband full-wave transmission response, and free-space measurements of an aperture of 29.7 × 20.9 cm² validate the polarization-selective behavior. The demonstrated response supports passive circular-polarization-selective receiver front ends and provides a polarimetric sensing platform for non-contact CFRP assessment.

## Introduction

Polarization control is a fundamental requirement in modern microwave systems, including satellite communication links, polarization-diverse antenna designs, radar front ends, synthetic aperture radar (SAR), and electromagnetic (EM) sensing platforms. In these systems, compact and passive structures capable of selectively manipulating polarization states and controlling wave propagation are highly desirable, as they enhance polarization discrimination, mitigate undesired coupling, and reduce overall system complexity. Planar metasurfaces, composed of subwavelength resonant elements arranged in two-dimensional arrays, provide an effective platform for achieving such functionalities. Owing to their low-profile configuration, compact footprint, and compatibility with standard microwave fabrication processes, metasurfaces enable flexible control of EM wave transmission, reflection, and polarization characteristics within a planar architecture.

In a circularly polarized wireless receiver, the desired signal can coexist with an oppositely handed interference component or with depolarized multipath generated by propagation and scattering. Although a circularly polarized antenna provides intrinsic polarization discrimination, its finite cross-polarization isolation can limit interference rejection. A passive polarization-selective surface positioned before the receiving antenna can provide an additional electromagnetic filtering stage by preferentially transmitting the desired handedness while attenuating the orthogonal circular-polarization component.

Beyond interference suppression, polarization-sensitive resonances can also serve as passive electromagnetic transducers for non-contact material sensing. When an anisotropic composite such as carbon-fiber-reinforced polymer (CFRP) is introduced into the metasurface-assisted interrogation region, variations in fiber orientation, laminate state, or internal defects perturb the resonant loading and can be detected through transmission, phase, and polarization-resolved signatures.

Chirality, arising from the absence of mirror symmetry in a structure, enables distinct electromagnetic (EM) responses to right-handed circularly polarized (RHCP) and left-handed circularly polarized (LHCP) waves. This polarization-dependent interaction leads to circular dichroism (CD), defined as the differential transmission or absorption between the two circular-polarization (CP) states. Although chiral EM effects were first observed in natural materials such as quartz^1^, their intrinsic response is typically weak and insufficient for practical microwave implementations. At microwave frequencies, realizing strong CD is particularly challenging due to the electrically large operating wavelengths, limited achievable geometric asymmetry within compact planar structures, and the inherently weak coupling between electric and magnetic resonant modes.

Chiral metasurfaces (CMSs) partially address these limitations by enabling cross-coupling between induced electric and magnetic responses at subwavelength scales, thereby enhancing polarization selectivity and enabling chiral transmission responses^2–4^. From a microwave design perspective, CMSs offer a planar and passive alternative to conventional polarization-selective components based on bulky waveguide structures or magnetically biased materials. However, achieving pronounced CD in transmission mode at multiple frequencies within a compact passive structure remains a nontrivial challenge.

A wide range of CMSs has been reported across microwave, terahertz, and infrared frequency regimes, demonstrating functionalities such as polarization conversion, asymmetric transmission (AT), electromagnetically induced transparency (EIT), and negative refractive index (NRI) behavior^5–11^. Within the microwave regime, however, metasurface designs that explicitly realize strong CD in transmission remain comparatively scarce. Most reported microwave metasurfaces primarily focus on polarization conversion or AT, exhibit relatively weak CD magnitude, and often operate either in reflection mode or at a single resonance frequency^12–17^.

In contrast, strong CD responses are more frequently reported at near-infrared, mid-infrared, and terahertz frequencies^18–27^, where deep subwavelength resonances, enhanced chiral coupling, and favorable material dispersion can be more readily exploited. However, the resonant mechanisms and material platforms employed at these higher frequencies differ fundamentally from those available at microwave frequencies. As a result, many of these approaches cannot be directly translated to planar, fabrication-compatible microwave CMSs.

Yamoun et al.^15^ reported a microwave CMS exhibiting CD based on extrinsic chirality introduced through sawtooth and zigzag configurations. In this design, the observed CD response arises primarily from the specific array arrangement and excitation conditions rather than from intrinsic asymmetry at the unit-cell level. The reported structure operates at a single resonance frequency and employs a geometrically complex three-dimensional (3D) configuration, which complicates physical interpretation and reduces compatibility with planar microwave implementations. Furthermore, the design does not demonstrate multi-frequency CD behavior with consistently high magnitude. Consequently, the combined realization of intrinsic unit-cell chirality, planar transmissive implementation, multi-frequency operation, and experimental validation remains insufficiently explored in existing microwave CMS designs.

Rotationally transformed bilayer chiral surfaces are well established. The author’s earlier study in ref. ^2^ used paired F-shaped resonators to produce Ku-band CD responses at 12.62 and 16.305 GHz with magnitudes of 0.5 and 0.4. Its preferred handedness changed from RHCP at the lower response to LHCP at the upper response. The present structure is not a frequency-scaled version of that design: it uses a single connected unequal-arm path on each metallic layer, and both modal maxima preferentially transmit the same propagation-relative RHCP state within one common X-band transmission window.

Here, a transmissive X-band chiral metasurface is developed using two controlled geometrical factors within one periodically repeated connected-strip unit cell. The compound front–back transformation removes the relevant mirror symmetry and establishes chiral interlayer coupling. The in-plane dimensional inequality w_1_ ≠ w_2_ makes the upper and lower terminal current paths electromagnetically inequivalent and enables the second pronounced modal contribution. Their separate and combined roles are established by the four design configurations.

The optimized surface produces local CD maxima of 0.72 and 0.75 at 9.672 and 10.636 GHz within a common RHCP transmission window, with CD = 0 at 10.3 GHz. The response is interpreted through propagation-relative circular states, Lorentz reciprocity, controlled configuration analysis, three-frequency surface-current distributions, and a broadband-validated equivalent-circuit model. Free-space measurements validate the forward response; the complete simulated circular coefficients are then translated into receiver-front-end metrics, and the polarization-sensitive modal response is further considered for antenna-interrogated CFRP sensing.

The principal contributions of this work are:

- A connected 11 × 11 mm² bilayer unit cell that realizes two forward-RHCP CD maxima of 0.72 and 0.75 at 9.672 and 10.636 GHz within one transmission window, with reciprocal backward-LHCP selectivity and a complete CD null at 10.3 GHz.
- A controlled two-factor design strategy that separates the origin of chirality from the origin of the second mode: the compound front–back transformation establishes chiral interlayer coupling, while w₁ ≠ w₂ creates electromagnetically inequivalent current paths without additional resonators or supercells.
- A reciprocity-consistent modal mechanism established through four controlled configurations and three-frequency surface-current analysis, identifying the lower even mode, the 10.3 GHz mixed transition, and the upper odd interlayer-coupled mode.
- A geometry-derived equivalent-circuit model validated over 8–12 GHz against full-wave samples.
- Experimental validation using a fabricated 27 × 19 array, together with desired-RHCP insertion losses of 1.17 and 0.57 dB, polarization extinction ratios of 9.55 and 7.47 dB, and a polarimetric sensing framework for non-contact CFRP assessment.

## Results

### Operating principle

**Fig. 1:**
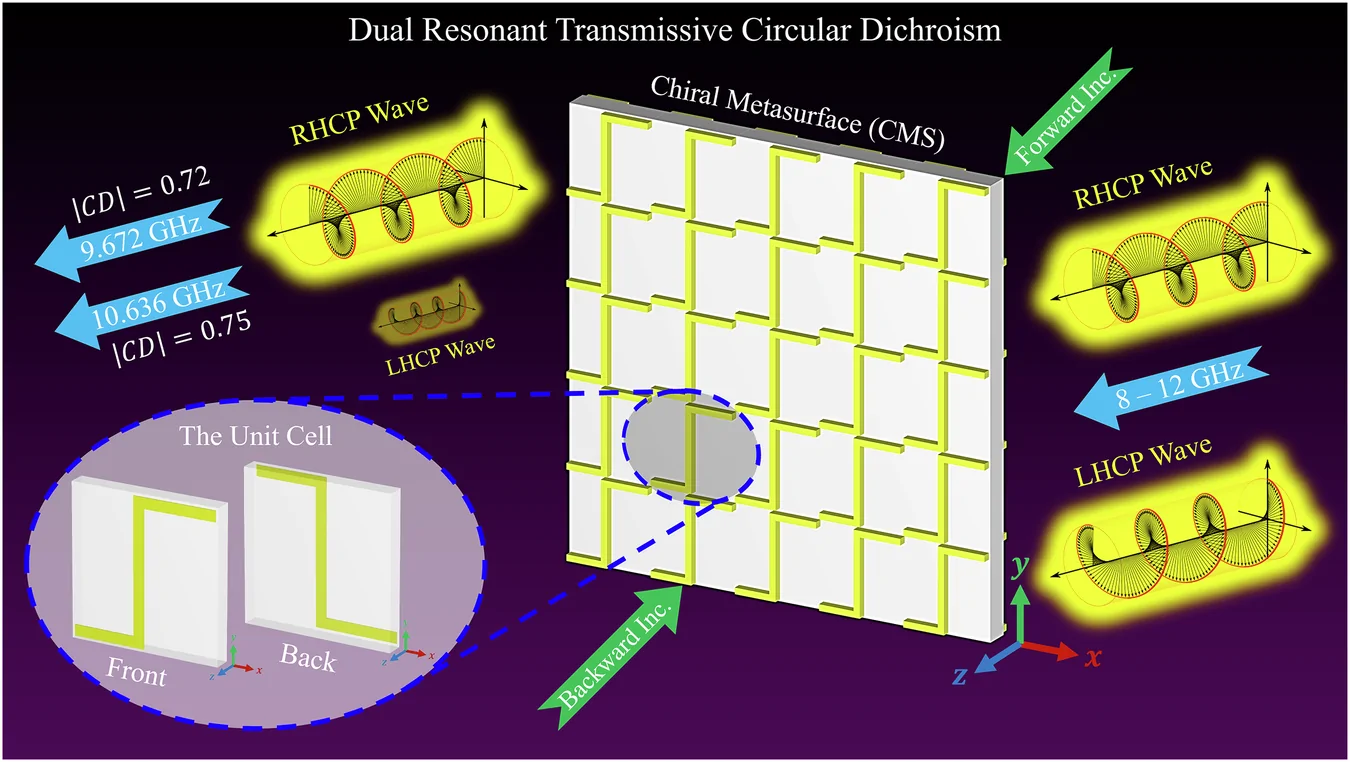
Reciprocal dual resonant circular-polarization-selective transmission of the chiral metasurface. The schematic shows the connected bilayer chiral metasurface and an enlarged front-and-back view of the unit cell under forward and backward illumination. Yellow metallic traces denote the patterned copper layers, the white slab denotes the dielectric substrate, green arrows indicate the propagation directions, and the yellow helical field illustrations indicate right-handed circularly polarized (RHCP) and left-handed circularly polarized (LHCP) waves. The blue arrows identify the two circular-dichroism (CD) maxima at 9.672 and 10.636 GHz, with absolute CD values of 0.72 and 0.75, respectively, and the 8–12 GHz analysis range. The coordinate triad defines the x, y, and z axes used throughout the electromagnetic analysis.

The operating principle is described in the propagation-relative circular basis. The compound front–back transformation establishes the chiral bilayer interaction, whereas terminal-arm inequality separates the two longitudinal current paths spectrally. Their combination produces the lower predominantly symmetric mode at 9.672 GHz and the upper predominantly antisymmetric interlayer-coupled mode at 10.636 GHz, separated by the 10.3 GHz CD null shown schematically in Fig. 1.

A geometrically chiral surface cannot be superimposed on its mirror image by rotation and translation. Consequently, propagation-relative RHCP and LHCP excitations couple differently to the electric currents on the two metallic layers and to their interlayer magnetic response. In general, this interaction may produce three related effects: unequal co-polarized transmission amplitudes, corresponding to circular dichroism; unequal co-polarized transmission phases, corresponding to circular birefringence or polarization rotation; and conversion between opposite CP states. The dominant effect in the proposed structure is co-polarized amplitude selectivity, for which one incident handedness is preferentially transmitted while the opposite handedness is attenuated within the same propagation direction. The cross-polarized CP coefficients remain comparatively small at the two operating frequencies.

For forward incidence, the metasurface preferentially transmits RHCP waves at the two CD maxima of 9.672 and 10.636 GHz, while LHCP transmission is suppressed at those operating points. These frequencies should not be interpreted as two isolated transmission passbands: the desired RHCP response lies within one common transmission window. At 10.3 GHz, the co-polarized RHCP and LHCP transmission amplitudes become equal, producing CD = 0 and eliminating circular-polarization discrimination at the intermediate frequency.

A reciprocal counterpart is observed when the wave propagates in the backward direction. The metasurface suppresses propagation-relative RHCP while preferentially transmitting propagation-relative LHCP at the same two frequencies. Because CP handedness is defined relative to the direction of propagation, backward LHCP is the reciprocal counterpart of forward RHCP, while backward RHCP is the reciprocal counterpart of forward LHCP. The observed interchange therefore represents reciprocal direction-reversed circular-polarization selectivity and does not, by itself, establish asymmetric transmission.

The polarization behavior of the proposed CMS is analyzed in the CP basis using the Jones transmission matrix T, which relates the transmitted and incident field components as expressed in Eq. (1)^28^. The matrix is ordered as [E_−_, E_+_]^T^, where − and + denote propagation-relative LHCP and RHCP states, respectively. The first subscript of T_pq_ identifies the transmitted polarization p, whereas the second identifies the incident polarization q:

1 $${T}_{{circ}}=\left[\begin{array}{cc}{T}_{--} & {T}_{-+}\\ {T}_{+-} & {T}_{++}\end{array}\right]$$

Here, T_−−_ and T_++_ denote the co-polarized LHCP and RHCP transmission coefficients, respectively, while T_−+_ and T_+−_ represent conversion into the opposite circular-polarization state. The transmitted and incident field vectors are related through Eq. (2). The first subscript identifies the transmitted state and the second identifies the incident state; therefore, the co-polarized and converted contributions are not interchangeable when interpreting CD.

2 $${E}_{{circ}}^{t}={T}_{{circ}}{E}_{{circ}}^{i}$$

Within this basis, the total transmitted powers for propagation-relative RHCP and LHCP incidence are P_+_ = |T_++_|² + |T_−+_|² and P_− _= |T_−−_|² + |T_+−_|², respectively. The present paper reports the amplitude-based metric CD = |T_++_| − |T_−−_| to remain consistent with the microwave CD literature used for comparison. Because |T_−+_| and |T_+−_| are small at 9.672 and 10.636 GHz, this metric predominantly measures the co-polarized CP transmission contrast rather than opposite-handedness conversion. A nonzero CD therefore signifies different attenuation of the two incident CP states, whereas a difference only in their transmitted phases would represent circular birefringence rather than amplitude dichroism.

For the present passive, linear and time-invariant structure, Lorentz reciprocity relates the forward and backward mode-to-mode transmission coefficients. Because CP handedness is defined relative to the direction of propagation, forward RHCP is reciprocal to backward LHCP, whereas forward LHCP is reciprocal to backward RHCP^29,30^. Thus, the forward-backward interchange of the preferred propagation-relative handedness is a reciprocal CP response and does not independently establish asymmetric transmission. The proposed response is therefore classified as two resonant CD maxima with reciprocal handedness reversal.

### Geometry and bilayer transformation

The design process of the proposed periodic CMS begins with the configuration of a single unit cell implemented on a Rogers 3010 substrate, which has a relative permittivity of ε_r_ = 10.2 and a loss tangent of tanδ = 0.0022. The unit cell incorporates an asymmetric strip-based structure that enables strong polarization selectivity and pronounced CD at two frequencies.

**Fig. 2:**
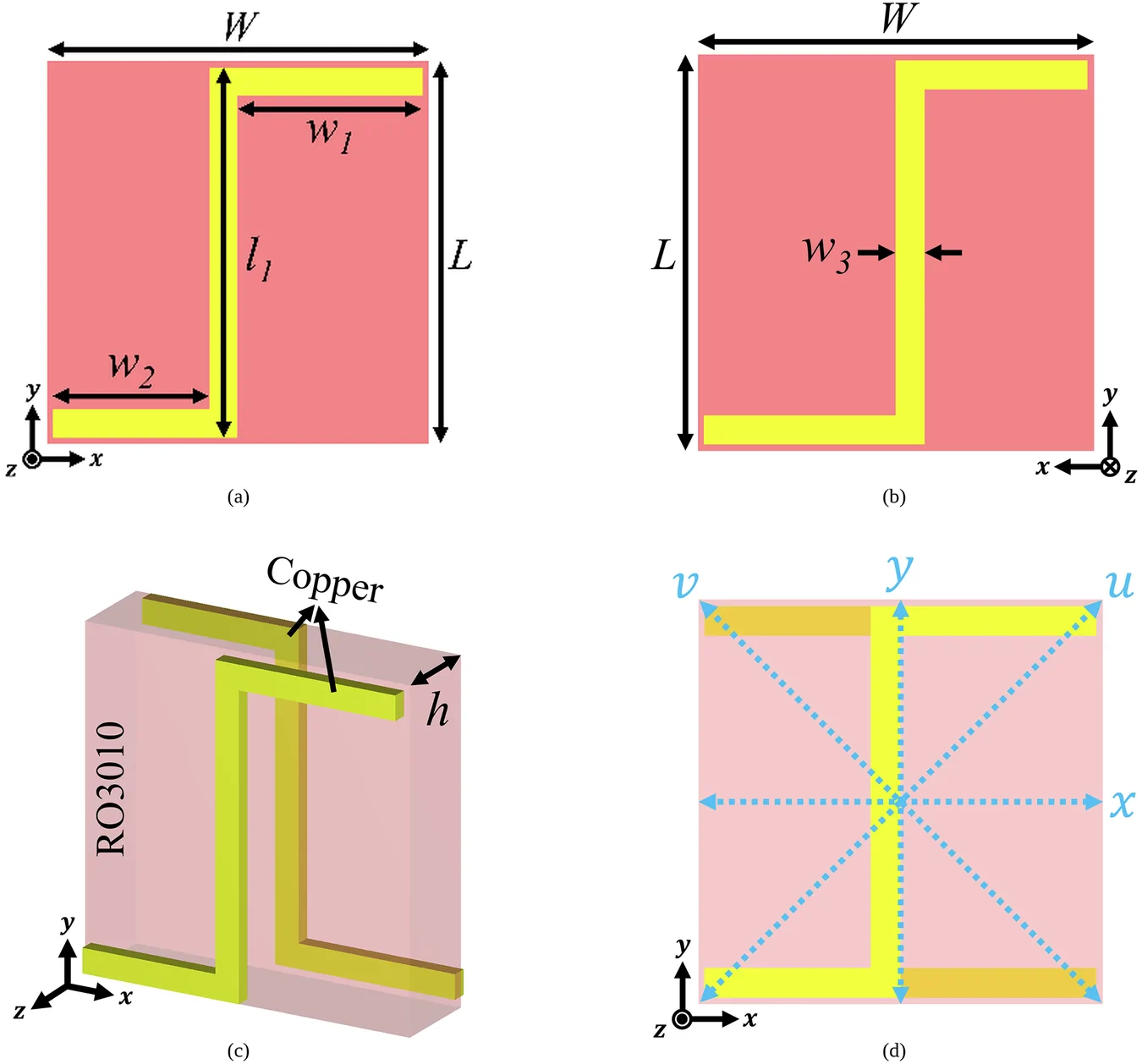
Geometry and controlled transformations of the bilayer unit cell. **a** shows the front metallic pattern and **b** shows the back metallic pattern. **c** presents the bilayer perspective view, including the Rogers 3010 substrate and copper layers. **d** identifies the geometrical axes used to define the controlled symmetry operations. The symbols L and W denote the unit-cell length and width, l_1_ denotes the principal vertical-strip length, w_1_ and w_2_ denote the unequal terminal-arm lengths, w_3_ denotes the strip width, and h denotes the substrate thickness. Yellow regions represent copper, the pink region represents the dielectric substrate, and the colored axes identify the x, y, z, u, and v directions used to describe the front–back transformation and in-plane path inequality.

The unit-cell design follows a systematic two-factor symmetry-engineering approach. As shown in Fig. 2a, the front side consists of one connected metallic path formed by a long vertical strip and two terminal horizontal arms. The vertical strip is laterally shifted, and the terminal arms are assigned unequal lengths w_1_ and w_2_. In contrast to the paired-F architecture of the authors’ previous work^2^ and the double-F topology in^5^, the second response is not introduced by adding a separate resonator or current branch. Instead, dimensional inequality within the same connected path creates two electromagnetically distinct current lengths. This in-plane factor is combined with a separately controlled rear-layer transformation.

The backside structure, illustrated in Fig. 2b, is derived by applying a 180° rotation about the y-axis followed by a 180° rotation about the z-axis before implementation on the opposite side of the substrate. This front-back relationship makes the complete bilayer arrangement chiral even for w_1_ = w_2_; therefore, the equal-arm configuration is not an achiral reference. In the final unequal-arm design, the compound transformation additionally strengthens the electric and magnetic coupling between the two inequivalent current paths, thereby enhancing the CP discrimination at both resonant responses.

A perspective view of the unit cell is presented in Fig. 2c, showing the dielectric substrate and the metallic patterns on opposite sides. The optimized dimensions required for fabrication are listed in Table 1.

**Table 1:** Optimized parameters of the proposed chiral metasurface unit cell

Parameter	Value (mm)	Parameter	Value (mm)
L, W	11	w_2_	4.5
l_1_	10.6	w_3_	0.8
w_1_	5.3	h	1.28

The square lattice period is *p* = 11 mm, and the dielectric thickness is *h* = 1.28 mm. At 9.672 and 10.636 GHz, p/λ₀ is 0.355 and 0.390, respectively, while h/λ₀ is 0.041 and 0.045. The maximum metallic dimension of 10.6 mm corresponds to 0.342λ₀ and 0.376λ₀. The structure is therefore treated as an electrically thin resonant periodic surface rather than as a deeply homogenized effective medium.

**Fig. 3:**
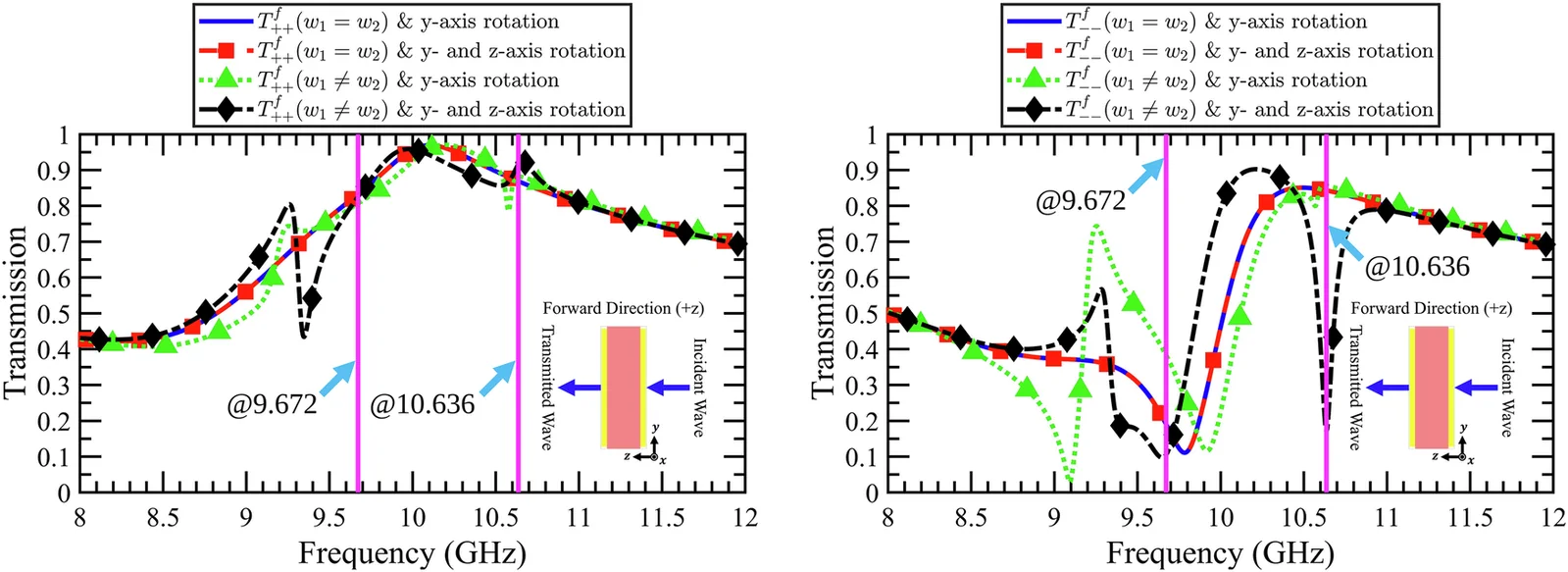
Controlled unit-cell configurations establish the origin of the two polarization-selective responses. **a** Shows the forward co-polarized RHCP transmission coefficient and **b** shows the forward co-polarized LHCP transmission coefficient from 8 to 12 GHz. RHCP and LHCP denote right- and left-handed circular polarization, respectively. The blue solid line represents equal terminal arms with the y-axis rotation; the red dash-dot line with square markers represents equal terminal arms with the compound y- and z-axis transformation; the green dotted line with triangle markers represents unequal terminal arms with the y-axis rotation; and the black dash-dot line with diamond markers represents unequal terminal arms with the compound transformation. The magenta vertical lines mark 9.672 and 10.636 GHz, and the cyan arrows identify the corresponding operating points. The insets define forward propagation along the positive z direction.

The strategic geometric modifications are highlighted using the x, y, u and v axes in Fig. 2d. The lateral displacement and unequal terminal-arm lengths define the current-path-inequality factor, whereas the combined y- and z-axis transformation defines the bilayer chiral-coupling factor. Treating these operations as separate variables enables their individual and combined effects to be evaluated through the four configurations shown in Figs. 3 and 5.

### Controlled configuration analysis and transmission response

The performance of the proposed CMS is evaluated under CP-wave excitation. In this analysis, an EM wave propagating in the +z direction is referred to as a forward wave, while an EM wave propagating in the −z direction is referred to as a backward wave. The handedness of a CP wave is defined with respect to its propagation direction. Consequently, the labels of a fixed electric-field rotation interchange when the propagation direction is reversed: forward RHCP and backward LHCP form a reciprocal pair, while forward LHCP and backward RHCP form the complementary reciprocal pair.

Figure 3 compares the forward co-polarized RHCP and LHCP transmission for four configurations: (1) equal terminal arms with y-axis rotation, (2) equal terminal arms with the compound y- and z-axis transformation, (3) unequal terminal arms with y-axis rotation, and (4) unequal terminal arms with the compound transformation. The equal-arm and unequal-arm labels refer only to the terminal dimensions and do not classify the complete bilayer as achiral or chiral.

For Configurations 1 and 2, where w_1_ = w_2_, the transformed bilayer remains chiral and exhibits one principal CD response, including the reported single-frequency response near 9.627 GHz. This confirms that terminal-arm inequality is not required for chirality or for the existence of CD. Applying the additional rear-layer transformation in Configuration 2 modifies and strengthens the chiral coupling, but it does not independently generate two pronounced responses. In Configuration 3, introducing w_1_ ≠ w_2_ makes the terminal current paths inequivalent and enables a second CP-selective contribution, although its discrimination remains weak. Configuration 4 combines this current-path inequality with the compound y- and z-axis transformation, producing the pronounced CD maxima at 9.672 and 10.636 GHz.

### Circular dichroism and common transmission window

**Fig. 4:**
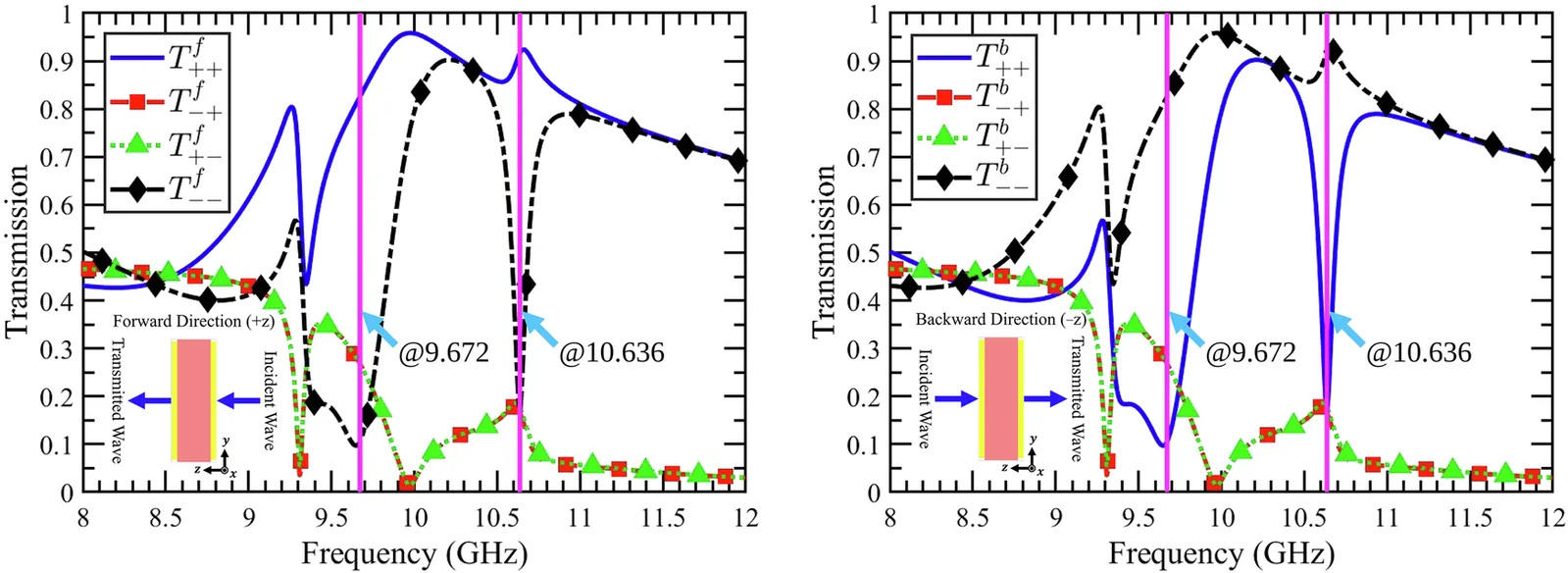
Forward and backward circular-basis transmission reveals reciprocal handedness reversal within one common transmission window. **a** presents the forward transmission coefficients and **b** presents the backward coefficients. In each panel, the blue solid line denotes the RHCP co-polarized coefficient T_++_, the black dashed line with diamond markers denotes the LHCP co-polarized coefficient T_−−_, and the red line with square markers and green dotted line with triangle markers denote the two opposite-handedness conversion coefficients T_−+_ and T_+−_, respectively. Superscripts f and b identify forward and backward propagation. The magenta vertical lines mark 9.672 and 10.636 GHz, while the cyan arrows identify the localized circular-dichroism extrema. The insets indicate the incident and transmitted propagation directions and the positive or negative z-axis convention.

Figure 4a shows one common forward-RHCP transmission window containing two local CD maxima at 9.672 and 10.636 GHz, with CD values of 0.72 and 0.75, respectively. The operating frequencies are identified from these local dichroism maxima, and the cross-polarized coefficients remain comparatively small at both frequencies.

The backward response in Fig. 4b exhibits the reciprocal interchange of the preferred propagation-relative handedness over the same common transmission window. The corresponding CD extrema occurs at 9.672 and 10.636 GHz, whereas the handedness discrimination vanishes at the intermediate frequency of 10.3 GHz.

The CD phenomenon of the proposed CMS is quantified using the co-polarized transmission-amplitude coefficients of RHCP and LHCP waves. The amplitude-based CD metric used for comparison with the referenced literature is defined in Eq. (3):

3 $${{CD}}^{f,b}=|{T}_{++}^{f,b}|-|{T}_{--}^{f,b}|$$

Here, CD_f_ and CD_b_ represent the forward and backward amplitude-based CD metrics, respectively. A nonzero value indicates different co-polarized transmission amplitudes for RHCP and LHCP waves within the same propagation direction. A positive value indicates preferential RHCP transmission, whereas a negative value indicates preferential LHCP transmission. This CD metric compares two different incident polarization states in one direction.

**Fig. 5:**
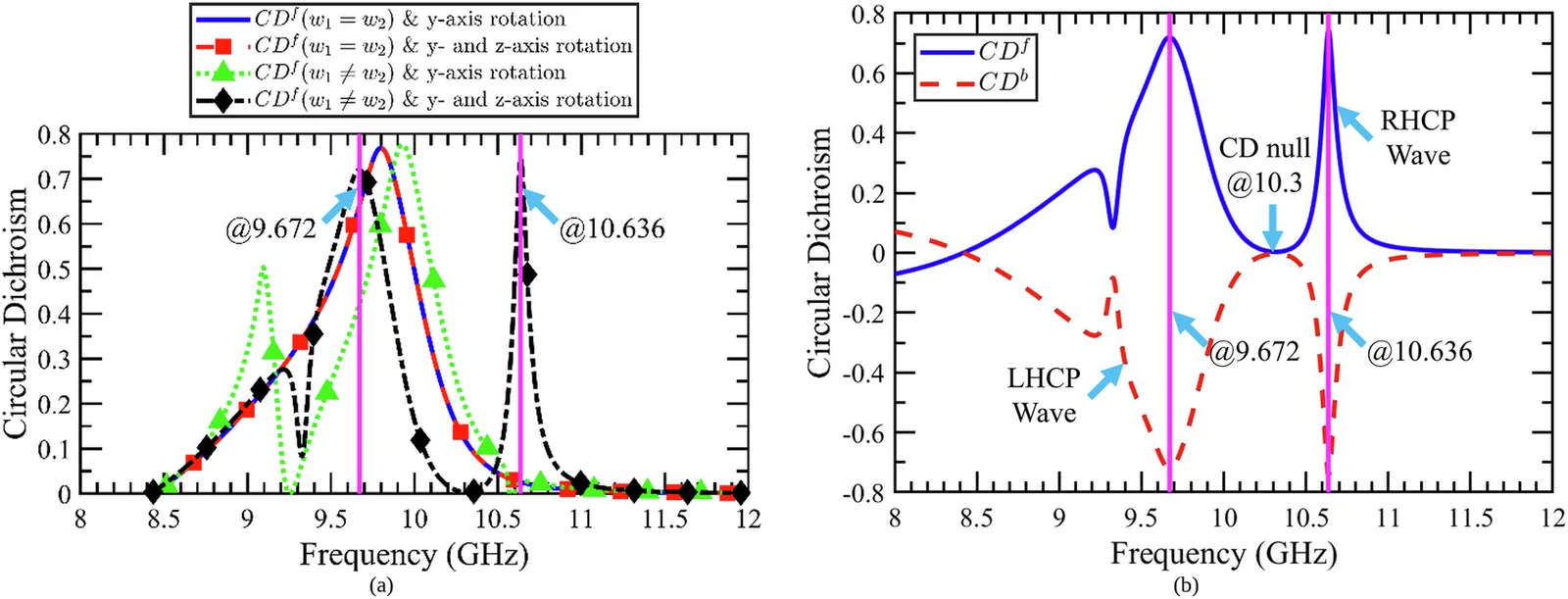
Circular-dichroism spectra show two localized maxima separated by an intermediate null. **a** Compares the forward circular-dichroism (CD) response of the four controlled unit-cell configurations. The blue solid line represents equal terminal arms with the y-axis rotation; the red line with square markers represents equal terminal arms with the compound y- and z-axis transformation; the green dotted line with triangle markers represents unequal terminal arms with the y-axis rotation; and the black dash-dot line with diamond markers represents unequal terminal arms with the compound transformation. **b** Shows the optimized forward CD as a blue solid line and backward CD as a red dashed line. The magenta vertical lines identify the CD extrema at 9.672 and 10.636 GHz, the cyan arrows indicate the associated RHCP and LHCP selective responses, and the cyan downward arrow marks the CD null at 10.3 GHz.

Figure 5a confirms that the transformed equal-arm bilayer supports one dominant CD response, whereas introducing w₁ ≠ w₂ enables the second modal contribution. Combining the unequal arms with the compound transformation produces the two pronounced CD maxima of the optimized design.

The optimized forward and backward CD spectra are shown in Fig. 5b. The forward response reaches local maxima of +0.72 and +0.75 at 9.672 and 10.636 GHz, respectively, whereas the backward spectrum exhibits the reciprocal values −0.72 and −0.75. Between these extrema, the CD decreases to exactly zero at 10.3 GHz because the forward co-polarized RHCP and LHCP transmission amplitudes become equal. The structural geometry remains chiral at this frequency; it is the frequency-dependent electromagnetic discrimination between the two CP states that vanishes. Consequently, the structure is described as exhibiting two localized CD maxima within a common transmission window, rather than broadband CD.

### Surface-current mechanism

**Fig. 6:**
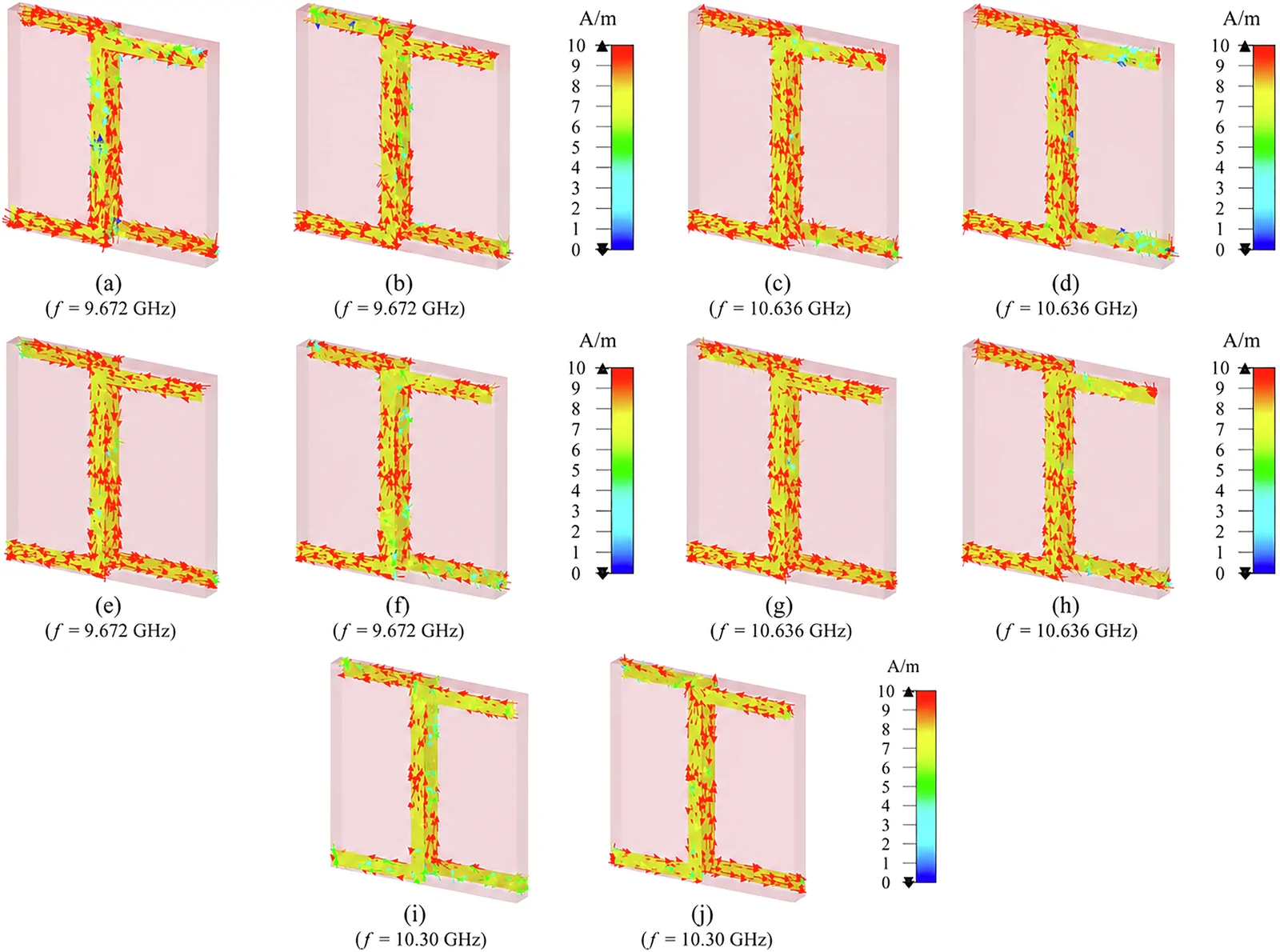
Surface-current distributions identify the lower even mode, upper odd mode, and intermediate circular-dichroism null. **a**–**d** Show forward excitation at the two circular-dichroism maxima: **a** and **b** correspond to RHCP and LHCP incidence at 9.672 GHz, and **c** and **d** correspond to RHCP and LHCP incidence at 10.636 GHz. **e**–**h** Show the reciprocal backward cases at the same frequencies: **e** and **f** correspond to RHCP and LHCP at 9.672 GHz, and **g** and **h** correspond to RHCP and LHCP at 10.636 GHz. **i** and **j** Show forward RHCP and LHCP excitation at the 10.30 GHz discrimination null. RHCP and LHCP denote right- and left-handed circular polarization. All panels use the same surface-current-density scale from 0 to 10 amperes per meter; dark blue indicates the lowest magnitude and red indicates the highest magnitude, with cyan, green, and yellow representing intermediate values.

To elucidate the modal origin of the two CD maxima and the intermediate discrimination null, the surface-current distributions on the top and bottom metallic layers were analyzed under CP excitation at 9.672, 10.3 and 10.636 GHz. Figure 6a–h shows the forward and backward reciprocal current pairs at the two CD maxima, while Fig. 6i, j shows the forward RHCP and LHCP currents at the 10.3 GHz CD null.

At 9.672 GHz under forward RHCP excitation [Fig. 6a], the currents on the top and bottom layers are predominantly parallel along the terminal arms, indicating an approximately in-phase symmetric configuration. This reduces the voltage differential across the overlapping layers and weakens excitation of the interlayer capacitance. The response is therefore dominated by the even mode and a strong electric-dipole contribution. The complementary LHCP case [Fig. 6b] contains a less coherent current arrangement and produces weaker co-polarized transmission.

At 10.636 GHz, the dominant current relationship changes. Under forward RHCP excitation [Fig. 6c], the corresponding currents on the top and bottom layers are predominantly antiparallel, producing an out-of-phase antisymmetric configuration. The resulting voltage differential strongly excites the interlayer capacitance and enhances the magnetic and interlayer-coupling response. The complementary LHCP case [Fig. 6d] couples less effectively to this preferred co-polarized transmission channel.

The backward cases demonstrate the reciprocal interchange of the current distributions associated with the propagation-relative CP states. The strong forward-RHCP current configuration at 9.672 GHz [Fig. 6a] corresponds to backward LHCP [Fig. 6f], while forward LHCP [Fig. 6b] corresponds to backward RHCP [Fig. 6e]. The same reciprocal pairing is observed at 10.636 GHz between Fig. 6c, h, and between Fig. 6d, g.

### Origin of the intermediate circular-dichroism null

At the intermediate frequency of 10.3 GHz, forward RHCP and LHCP excitations produce closely comparable surface-current distributions on the front and back metallic layers, as shown in Fig. 6i, j. The similar current localization and magnitude indicate that the two incident circular-polarization states excite the unit cell with nearly equal overall strength. Unlike the two CD maxima, where one handedness couples preferentially to the corresponding resonant mode, neither RHCP nor LHCP experiences dominant modal coupling at 10.3 GHz. The resulting front–back current responses therefore provide no net electromagnetic preference for either incident handedness. This balanced excitation is consistent with the equality of the co-polarized circular-transmission amplitudes, $|{T}_{++}^{f}|=|{T}_{--}^{f}|$ and consequently, ${{CD}}_{\left(10.3{GHz}\right)}^{f}=|{T}_{++}^{f}|-|{T}_{--}^{f}|=0$. The metasurface remains geometrically chiral at this frequency; however, its frequency-dependent discrimination between RHCP and LHCP vanishes, producing the observed CD null.

### Equivalent-circuit model and broadband validation

To further interpret the two modal CD maxima revealed by the surface-current distributions, an equivalent circuit model of the proposed unit cell is developed to represent the coupling between the top and bottom layers. The model contains the even and odd resonant branches associated with the 9.672 and 10.636 GHz extrema. The 10.3 GHz CD null is not treated as a separate circuit resonance; it is the spectral transition at which the net CP discrimination of the two modal contributions vanishes.

**Fig. 7:**
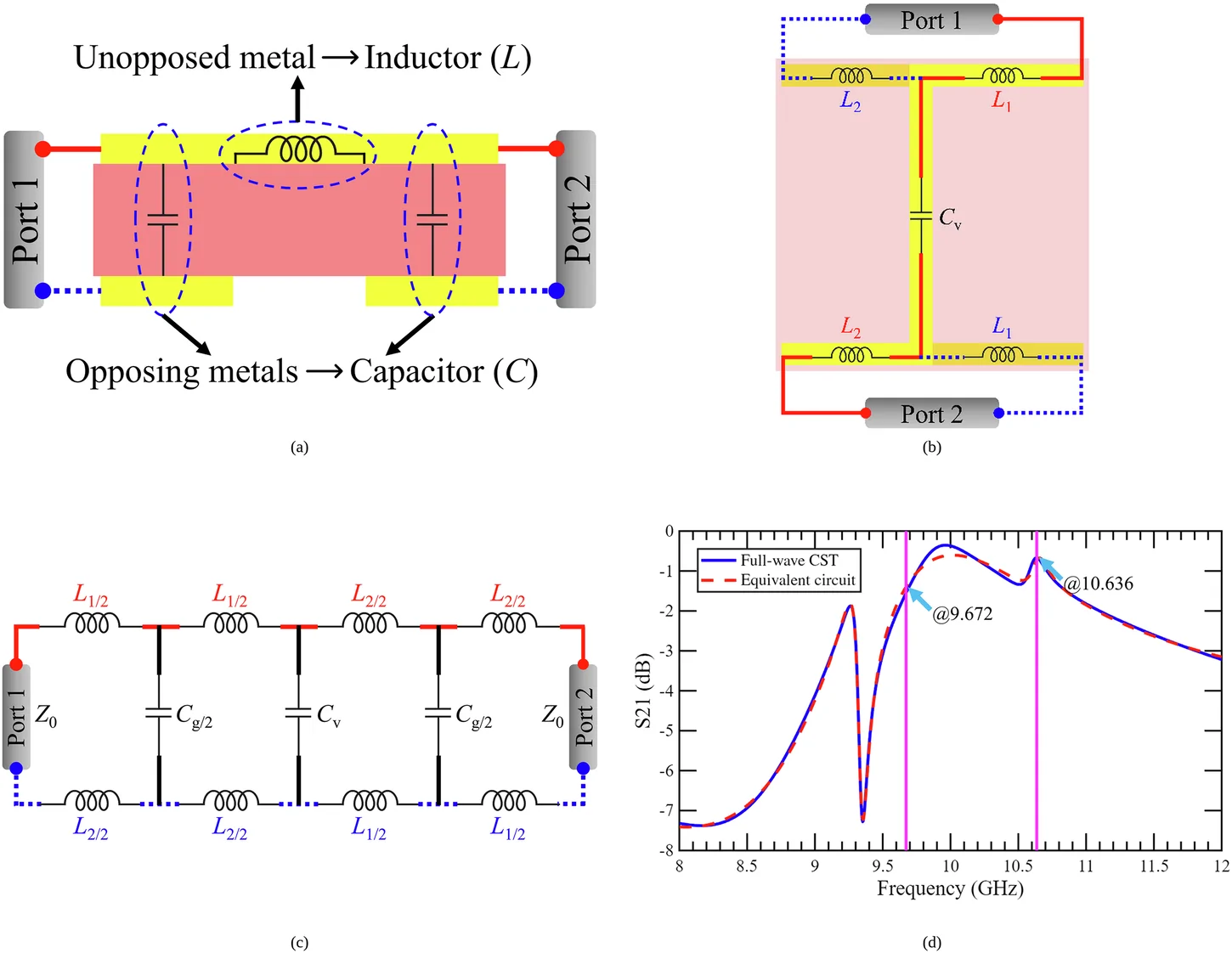
Geometry-derived equivalent-circuit representation reproduces the broadband full-wave transmission response. **a** Maps the overlapping and non-overlapping metallic regions to capacitive and inductive elements, respectively. The red solid trace denotes the top metallic layer, the blue dotted trace denotes the bottom layer, the yellow regions indicate non-opposed metal associated with inductance, and the overlapping pink region indicates capacitive coupling. **b** Maps the unit-cell geometry to the longitudinal inductances L_1_ and L_2_ and the interlayer capacitance C_v_ between Port 1 and Port 2. **c** Shows the complete lumped network, including the split inductances, interlayer coupling, fringe capacitances, and port reference impedance Z_0_. **d** Compares the full-wave CST Studio Suite result as a blue solid line with the equivalent-circuit prediction as a red dashed line for the transmission coefficient S_21_ in decibels. Magenta vertical lines mark 9.672 and 10.636 GHz, and cyan arrows identify the two modal regions.

We use the equivalent circuit model of the proposed unit cell to analyze the structure, and the observed dual resonant response is achieved from this model. In Fig. 7a, the mapping from geometry to circuit is defined: solid red traces denote the top metal layer, dotted blue traces denote the bottom layer, and Port 1/Port 2 excite and terminate both layers in parallel. Metal regions that do not face metal on the opposite layer support longitudinal current and are modeled as inductors (*L*), whereas regions where the two layers face across the substrate are modeled as capacitors (*C*). Figure 7b applies this mapping to the unit cell: two inductive paths connect Port 1 to Port 2 for the top layer, the upper horizontal arm is modeled by ${L}_{1}$ and the lower arm by ${L}_{2}$; for the bottom layer, the upper horizontal arm is modeled by ${L}_{2}$ and the lower by ${L}_{1}$. The mid-points of these paths are coupled by the interlayer capacitor ${C}_{v}$. A small shunt-to-ground capacitance ${C}_{g}$ at the mid-points represents pad/edge fringing present in the physical layout. The complete LC network corresponding to this mapping is shown in Fig. 7c. It represents the simplified lumped configuration used for circuit-level analysis and simulation. Each layer is modeled as a separate inductive branch between Port 1 and Port 2, with inductors ${L}_{1}/2$ and ${L}_{2}/2$ placed in series along the top and bottom branches, respectively. The mid-nodes of these branches are coupled by the interlayer capacitor ${C}_{v}$, while small capacitors ${C}_{g}/2$ are connected from each mid-node to ground to represent fringing and pad loading. This configuration captures the EM coupling between the top and bottom metallic layers and reproduces the dual resonant behavior observed in the full-wave simulations.

The horizontal arm inductances are obtained from the flat-wire expression given by Eq. (4) ^31^:

4 $${L}_{1,2}=\frac{{\mu }_{0}l}{2\pi }\left[{\mathrm{ln}}\left(\frac{2l}{w+t}\right)+0.5+0.2235\left(\frac{w+t}{l}\right)\right]$$

Here, *l*, *w*, and *t* are the strip length, width, and thickness, respectively, and ${\mu }_{0}=4\pi \times {10}^{-7}{\rm{H}}/{\rm{m}}$. Using the unit cell line dimensions yields ${L}_{1}=3.261{\rm{nH}}$ and ${L}_{2}=2.627{\rm{nH}}$.

The interlayer capacitance from the vertical overlap is modeled as a parallel plate capacitor^32^:

5 $${C}_{v}={\varepsilon }_{0}{\varepsilon }_{r}\frac{A}{h}$$

Here, *A* is the overlap area, *h* is the substrate thickness, ${\varepsilon }_{0}=8.854\times {10}^{-12}{\rm{F}}/{\rm{m}}$, and ${\varepsilon }_{r}$ is the relative permittivity. The parallel-plate estimate gives ${C}_{v}=0.598{\rm{pF}}$; a modest fringe correction of ≈3% yields ${C}_{v}=0.616{\rm{pF}}$.

In operation, the two branches support distinct longitudinal current configurations. Under odd (antisymmetric) excitation, the mid-node voltages on the two layers are equal in magnitude and opposite in sign, producing a strong differential across *C*_*v*_(the *C*_*v*_-dominated mode). Under even (symmetric) excitation, the mid-node voltages are nearly equal, the voltage across ${C}_{v}$ is minimal, and the resonance is directed by the small shunt ${C}_{g}$ to ground (the *C*_*g*_-dominated mode). These two cases are represented by the following reductions of the split branches: ${L}_{-}={L}_{1}{L}_{2}/[4({L}_{1}+{L}_{2})]$ and ${L}_{+}=({L}_{1}+{L}_{2})/4$. Here, ${L}_{-}$ corresponds to the inductive parameter of the antisymmetric configuration that drives the interlayer overlap, and ${L}_{+}$ to the symmetric configuration in which the overlap is effectively dark. Both resonances follow the relation given by Eq. (6):

6 $${f}_{\!\pm }=\frac{1}{2\pi \sqrt{{L}_{{eff},\pm }{C}_{{eff},\pm }}}$$

The geometry-derived modal reductions give Lₑ = 1.472 nH and Cₑ = 0.184 pF for the lower even mode, and Lₒ = 0.364 nH and Cₒ = 0.616 pF for the upper odd mode. These values fix the two principal modal frequencies at 9.672 and 10.636 GHz, respectively. For broadband validation, the LC topology of Fig. 7c was expressed in modal form and extended only by physically required non-ideal terms: finite modal damping, the non-resonant direct transmission path through the aperture and substrate, and a weak fringe-associated parasitic term. The last term accounts for the narrow pre-resonant feature near 9.35 GHz and is associated with edge/fringing loading represented by C_g_.

The broadband equivalent-circuit model (ECM) transfer function was written as the coherent sum of a slowly varying direct path and damped modal terms:

7 $${S}_{21,{ECM}}\left(f\right)={H}_{d}\left(f\right)+\sum _{m\in \left\{p,e,o\right\}}\frac{{k}_{m}}{1+{{jQ}}_{m}\left(f/{f}_{\!m}-{f}_{\!m}/f\right)}$$

The principal frequencies were fixed at fₑ = 9.672 GHz and fₒ = 10.636 GHz by the geometry-derived L and C values. A global least-squares fit to CST samples gave Qₑ = 6.22 and Qₒ = 73.48, corresponding to Rₑ = 14.39 Ω and Rₒ = 0.33 Ω. The weak fringe-associated term occurs at f_p_ = 9.318 GHz with Q_p_ = 86.25; only damping, coupling and direct-path coefficients were fitted.

Loss is incorporated through the finite modal quality factors rather than neglected. The modal resistances Rₑ and Rₒ reported above are total effective series damping terms. They include dielectric dissipation, finite copper conductivity, radiation leakage, and distributed parasitic effects that are not represented individually by the ideal LC topology. To quantify the dielectric contribution explicitly, each modal capacitance is treated as a low-loss capacitor with the RO3010 loss tangent tanδ = 0.0022. Its equivalent series dielectric resistance is R_diel_ = tanδ/(2πfC). This gives R_diel,e_ = 0.197 Ω for Cₑ = 0.184 pF at 9.672 GHz and R_diel,o_ = 0.053 Ω for Cₒ = 0.616 pF at 10.636 GHz. The remaining effective damping terms are therefore approximately 14.19 Ω and 0.277 Ω for the even and odd modes, respectively, and represent the combined conductor, radiative, coupling, and omitted distributed losses. This decomposition does not alter the previously obtained broadband response because the total fitted modal resistances remain Rₑ = 14.39 Ω and Rₒ = 0.33 Ω.

Figure 7d compares the broadband prediction of the lossy modal equivalent-circuit model directly with the CST forward-RHCP co-polarized transmission magnitude from 8 to 12 GHz. The comparison therefore tests the complete transmission envelope, the narrow pre-resonant dip, the two principal modal regions, and the out-of-band roll-off.

### Experimental validation

**Fig. 8:**
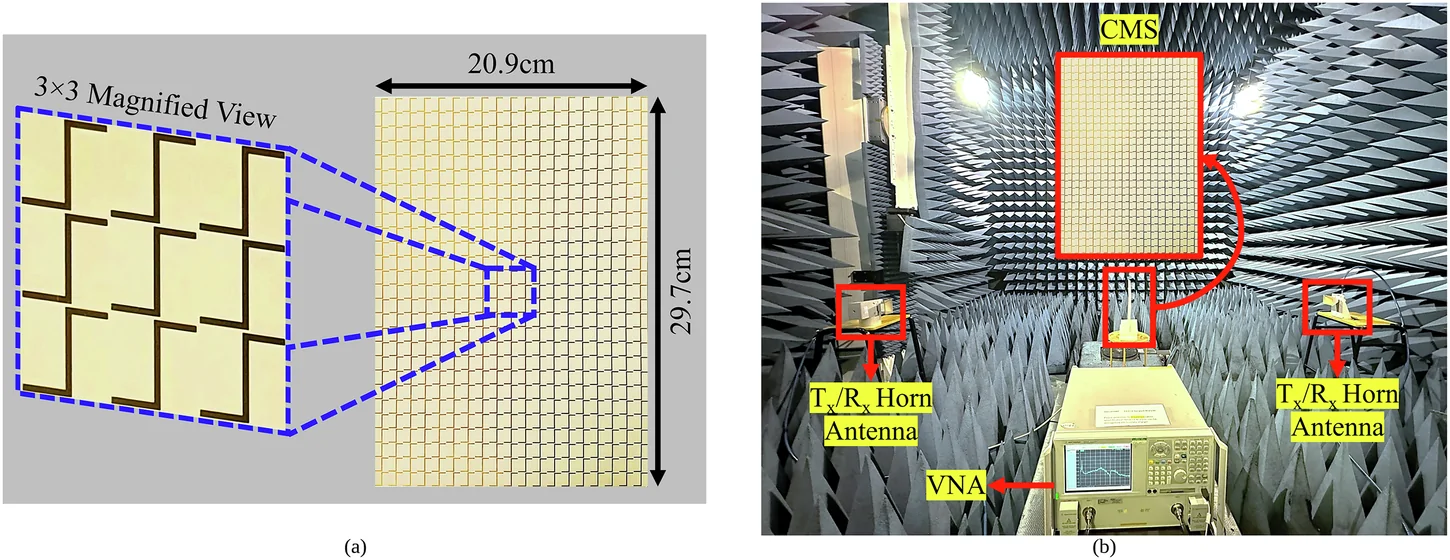
Fabricated chiral-metasurface array and free-space measurement arrangement. **a** Shows the 27 by 19 unit-cell prototype, its 29.7 by 20.9 centimeter aperture, and an enlarged 3 by 3 cell region. **b** Shows the free-space measurement configuration inside the anechoic chamber. The chiral metasurface is centered between the transmitting and receiving X-band horn antennas and connected through the measurement path to a vector network analyzer (VNA). CMS denotes chiral metasurface, T/R denotes transmitting or receiving, and VNA denotes vector network analyzer. Red outlines identify the metasurface and measurement components, yellow labels identify the equipment, and the red curved arrow indicates the transmission path through the sample.

To validate the simulated results, a 27 × 19 prototype containing 513-unit cells was fabricated with an overall aperture of 29.7 × 20.9 cm², as shown in Fig. 8a. The electrically large, odd-cell aperture provides a physical center cell for alignment and represents a practical balance among finite-aperture performance, fabrication and measurement constraints. The array size does not determine the unit-cell resonances. An experimental demonstration of the fabricated CMS was conducted in an anechoic chamber to measure its transmission response under a linearly polarized (LP) incident wave. The measurement setup, illustrated in Fig. 8b, comprised two identical LP horn antennas operating in the X-band, along with a vector network analyzer (VNA). One horn antenna functioned as the transmitting source, while the other served as the receiving antenna, positioned on opposite sides of the metasurface with the CMS placed at the center.

For the experimental validation reported here, the co-polarized and cross-polarized transmission coefficients for forward x- and y-polarized LP incidence were measured by appropriately orienting the two horn antennas, as shown in Fig. 8b. The measured forward LP transmission coefficients were subsequently converted into forward CP transmission coefficients using Eq. (8) ^28^. The backward CP response discussed in the numerical analysis is interpreted using the same propagation-relative handedness convention and the reciprocal pairing of the corresponding polarization states.

8 $$\left[\begin{array}{cc}{T}_{++} & {T}_{+-}\\ {T}_{-+} & {T}_{--}\end{array}\right]=\frac{1}{2}\left[\begin{array}{cc}{T}_{{xx}}+{T}_{{yy}}+i\left({T}_{{xy}}-{T}_{{yx}}\right) & {T}_{{xx}}-{T}_{{yy}}+i\left({T}_{{xy}}+{T}_{{yx}}\right)\\ {T}_{{xx}}-{T}_{{yy}}-i\left({T}_{{xy}}+{T}_{{yx}}\right) & {T}_{{xx}}+{T}_{{yy}}-i\left({T}_{{xy}}-{T}_{{yx}}\right)\end{array}\right]$$

**Fig. 9:**
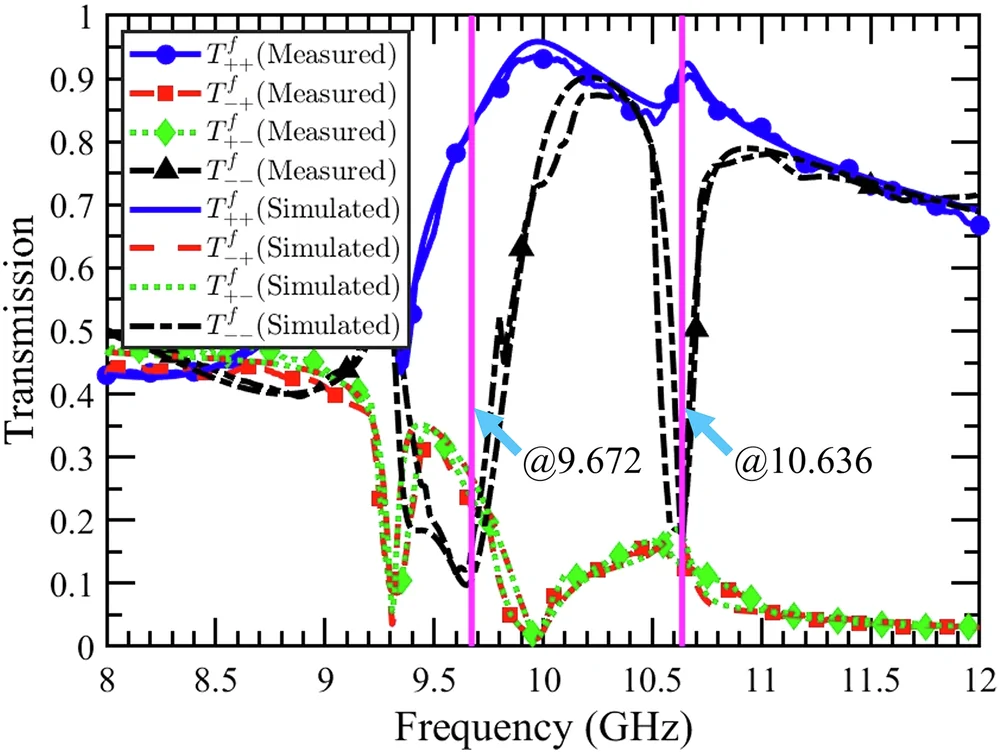
Measured and simulated forward circular-basis transmission coefficients validate the polarization-selective response. The four forward coefficients are shown from 8 to 12 GHz. Circular markers and other discrete markers denote measured data, while continuous or dashed lines denote the corresponding full-wave simulation. Blue denotes the RHCP co-polarized coefficient T_++_, black denotes the LHCP co-polarized coefficient T_−−_, red denotes the conversion coefficient T_−+_, and green denotes the conversion coefficient T_+−_. RHCP and LHCP denote right- and left-handed circular polarization. The magenta vertical lines identify 9.672 and 10.636 GHz, and the cyan arrows mark the localized polarization-discrimination extrema. Transmission is plotted as a linear magnitude.

Figure 9 compares the measured and simulated forward circular-basis transmission coefficients. The experiment reproduces the common desired-polarization transmission window and the two localized discrimination extrema. The remaining frequency and magnitude differences are consistent with fabrication tolerances, dielectric property variation, finite-aperture effects and alignment uncertainty. This agreement verifies that the modal response predicted for the periodic unit cell persists in the fabricated finite array.

### Receiver-front-end performance metrics and polarization-selective response

**Fig. 10:**
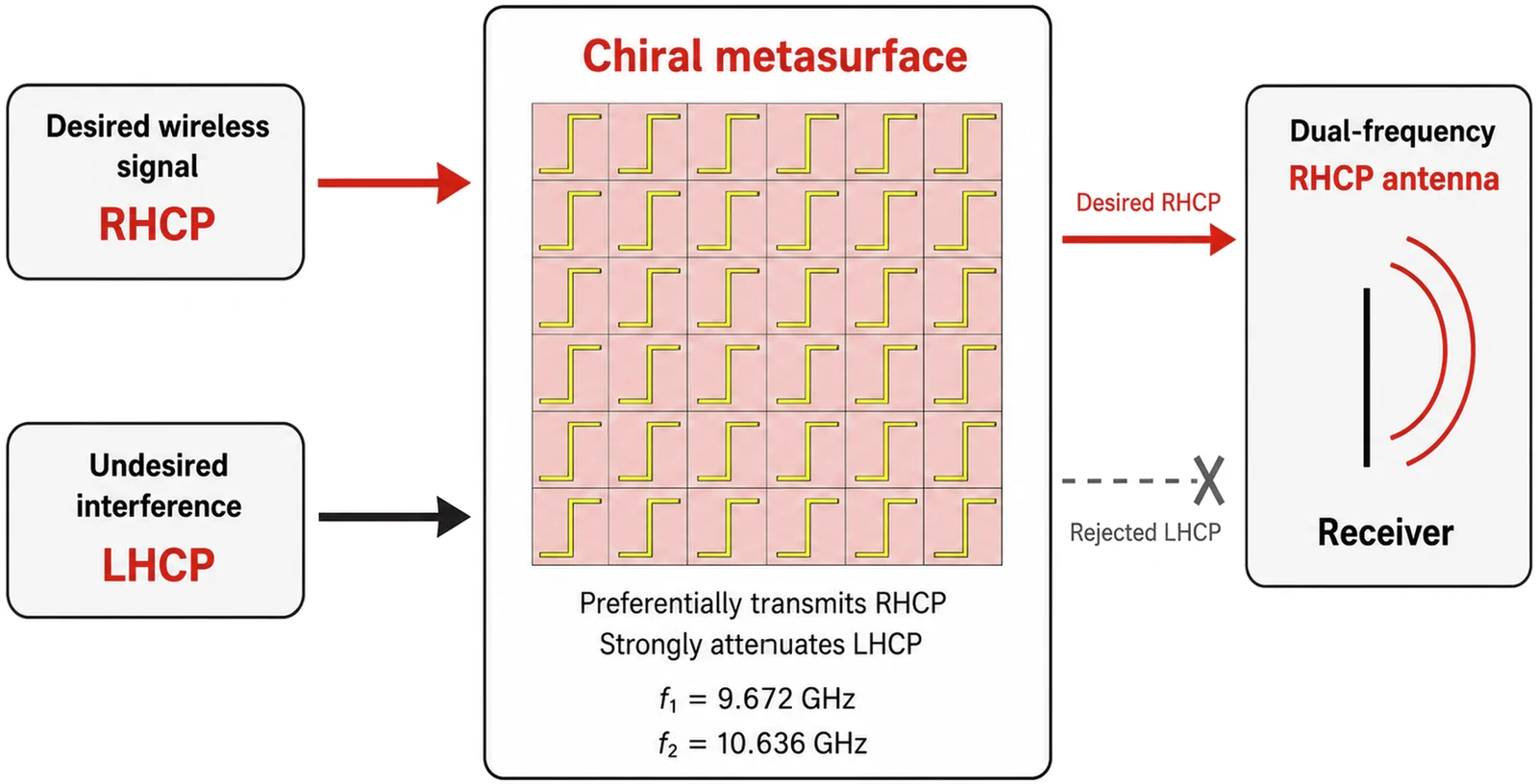
Passive circular-polarization-selective receiver-front-end concept. The schematic shows a desired RHCP wireless signal and an undesired LHCP interference component incident on the chiral metasurface before a dual-frequency RHCP receiving antenna. RHCP and LHCP denote right- and left-handed circular polarization, respectively. The red arrow represents preferential transmission of the desired RHCP component, the black arrow represents the incident LHCP interference, and the gray dashed path ending at the cross mark represents rejection of the undesired component before the receiver. The central patterned panel represents the chiral metasurface operating at 9.672 and 10.636 GHz, and the antenna symbol represents the downstream circularly polarized receiver.

The experimentally validated polarization-selective response can be interpreted as a passive receiver-front-end function at the two CD maxima. As illustrated in Fig. 10, the metasurface is positioned before an RHCP receiving antenna and preferentially transmits the desired RHCP component at 9.672 and 10.636 GHz while attenuating an LHCP interference component. The intermediate frequency of 10.3 GHz is not treated as an operating point because the CD and the associated polarization extinction vanish there.

9 $${\eta }_{R}={|{T}_{++}^{f}|}^{2}+{|{T}_{-+}^{f}|}^{2}$$

10 $${\eta }_{L}={|{T}_{--}^{f}|}^{2}+{|{T}_{+-}^{f}|}^{2}$$

The desired-state insertion loss, rejected-polarization attenuation, and polarization extinction ratio (PER) are then calculated as:

11 $${{IL}}_{R}=-10{log }_{10}\left({\eta }_{R}\right)$$

12 $${A}_{L}=-10{log }_{10}\left({\eta }_{L}\right)$$

13 $${\rm{PER}}=10{log }_{10}\left(\frac{{\eta }_{R}}{{\eta }_{L}}\right)$$

For a desired RHCP signal and an LHCP interference signal, the output signal-to-interference ratio (SIR) is improved by the polarization extinction ratio, such that SIR_out_ = SIR_in_ + PER in decibels. At 9.672 GHz, η_R_ = 0.7638 and η_L_ = 0.0848, giving an RHCP insertion loss of 1.17 dB, an LHCP attenuation of 10.72 dB and a PER of 9.55 dB. At 10.636 GHz, η_R_ = 0.8762 and η_L_ = 0.1570, corresponding to 0.57 dB insertion loss, 8.04 dB attenuation and 7.47 dB PER. These metrics are summarized in Table 2.

**Table 2:** Receiver-front-end metrics derived from forward transmission coefficients

Metric	9.672 GHz	10.636 GHz
η_R_	0.7638	0.8762
η_L_	0.0848	0.1570
IL_R_ (dB)	1.17	0.57
A_L_ (dB)	10.72	8.04
PER (dB)	9.55	7.47

The metasurface therefore acts as a passive pre-antenna spatial filter at two localized X-band operating frequencies. Potential applications include circularly polarized receiver front ends, polarization-selective radar and sensing apertures, and suppression of oppositely handed interference before the antenna. Geometric scaling can translate the two-mode response to other application-specific frequencies, while selective changes to the unequal paths and overlap region can modify their separation.

### Polarimetric CFRP sensing and structural-health monitoring

The dual-resonant circular-polarization-selective response of the proposed chiral metasurface can be employed as a passive electromagnetic transducer for non-contact assessment of carbon-fiber-reinforced polymer (CFRP) laminates. In this configuration, transmitting and receiving antennas establish an over-the-air interrogation link, while the CFRP specimen is positioned within the metasurface-assisted sensing region at a controlled standoff distance. The metasurface enhances the interaction between the incident electromagnetic field and the material under test, converting changes in the laminate condition into measurable spectral and polarimetric variations in the received transmission response.

CFRP exhibits anisotropic microwave behavior because its effective conductivity, permittivity, and surface impedance depend on fiber orientation, laminate thickness, stacking arrangement, ply continuity, and the polarization of the interrogating field. Introducing a CFRP laminate into the sensing region modifies the electromagnetic loading of the metasurface and perturbs its surface-current distributions, interlayer coupling, and resonant modal response. These perturbations can appear as shifts in resonance frequency, changes in transmission magnitude and phase, variations in notch depth and bandwidth, and modifications of the co-polarized and cross-polarized transmission coefficients. The associated changes in ${\eta }_{R}$, ${\eta }_{L}$, rejected-polarization attenuation, and polarization extinction ratio provide additional polarization-resolved sensing observables beyond conventional magnitude-only interrogation.

Structural discontinuities such as delamination, matrix cracking, voids, internal channels, impact damage, and fiber breakage disturb the local conductive pathways and electromagnetic boundary conditions of the laminate. Consequently, the transmission signature measured for a damaged specimen differs from that of an intact reference condition. Variations in laminate thickness, fiber-layer state, or ply arrangement similarly modify the effective loading and can be identified through systematic changes in the resonant response. Because the proposed metasurface supports two distinct resonant modes, the two operating frequencies can provide complementary sensing channels, enabling the material condition to be represented by a multidimensional spectral–polarimetric signature rather than by a single resonance feature.

A chiral-metasurface-assisted implementation of this sensing principle has been demonstrated^33^, where antenna-based transmission measurements were used for CFRP fiber-layer assessment and internal-defect interrogation. The measured response showed that changes in the laminate state and defect condition produce observable resonance displacement, transmission-amplitude variation, and spectral redistribution. The proposed metasurface can therefore be incorporated into compact antenna-enabled readers, controlled-standoff scanning platforms, or remotely interrogated sensing nodes for couplant-free microwave non-destructive evaluation and structural-health monitoring of composite components used in aerospace, automotive, marine, energy, and other safety-critical engineering systems.

## Discussion

Table 3 compares the proposed metasurface with representative microwave chiral surfaces in terms of electrical size, transmission configuration, and circular-dichroism performance. The present 11 × 11 mm^2^ unit cell has ${p}_{\max }/{\lambda }_{0}=0.355$ and 0.390 at 9.672 and 10.636 GHz, respectively. Its period therefore remains below ${\lambda }_{0}/2$ in free space at both operating frequencies, although the structure is not deeply subwavelength within the high-permittivity substrate. Within the compared set, the proposed metasurface is the only design exhibiting two CD maxima with ∣${\rm{CD}}$∣≥0.7, while preferentially transmitting the same propagation-relative RHCP state at both operating points.

**Table 3:** Performance comparison with representative microwave chiral surfaces

Study	Region	Cell size and *p*_max_/λ_0_	Layers	Operating mode	CD magnitude; count | CD | ≥ 0.7
^2^	Microwave	11 × 11 mm²; 0.463, 0.598	2	Transmission	0.5, 0.4; 0
^3^	Microwave	NR; NR	3	Transmission	NR; NR
^12^	Microwave	10 × 10 mm²; 0.444–0.554	2	Transmission	0.719, 0.3; 1
^13^	Microwave	10 × 10 mm²; 0.294	2	Transmission	0.7; 1
^14^	Microwave	7 × 7 mm²; 0.560	2	Transmission	0.6; 0
^15^	Microwave	9 × 9 mm²; 0.273	2	Transmission	0.9; 1
^16^	Microwave	14 × 14 mm²; 0.234–0.756	2	Transmission	0.74, 0.64, 0.5; 1
^17^	Microwave	11.6 × 11.6 mm²; 0.466–0.659	2	Transmission	0.6, 0.6, 0.42; 0
This work	Microwave	11 × 11 mm²; 0.355, 0.390	2	Transmission	0.72, 0.75; 2

Previous multi-response chiral and polarization-converting metasurfaces commonly employ additional resonators, multiple current branches, or multilayer coupling arrangements to generate distinct spectral responses. More broadly, the representative designs in Table 3 demonstrate that increasing the number of polarization-selective responses often requires greater geometrical or structural complexity, while the achieved CD magnitudes are not necessarily maintained across all operating frequencies. In contrast, the present structure produces two pronounced same-handedness CD maxima using one connected unequal-arm current path on each metallic layer. The second response is therefore generated through controlled electromagnetic inequivalence within the existing current path rather than through the addition of a separate resonator, disconnected branch, or extra functional layer.

The response-generation mechanism is established through the controlled comparison of four geometrical configurations. The compound front–back transformation creates the chiral interlayer interaction and supports a dominant CD response even when the terminal arms are equal. Introducing ${w}_{1}\ne {w}_{2}$ makes the longitudinal current paths electromagnetically inequivalent and enables the second pronounced modal contribution. The surface-current distributions identify a lower predominantly symmetric mode at 9.672 GHz and an upper antisymmetric interlayer-coupled mode at 10.636 GHz. This physical interpretation is supported consistently by the equivalent-circuit model, broadband full-wave validation, and free-space measurements.

The proposed device should be interpreted as an electrically thin dual-resonant periodic surface rather than as a deeply homogenized, broadband, or angle-invariant medium. Circular-polarization discrimination vanishes at 10.3 GHz, where the co-polarized RHCP and LHCP transmission amplitudes become equal. Oblique incidence also progressively shifts and weakens the modal features outside the near-normal operating regime. These characteristics define the intended operating envelope without affecting the demonstrated dual resonant polarization discrimination.

Within this operating envelope, the measured response reproduces the two polarization-selective extrema predicted by the full-wave model. The complete circular-basis transmission coefficients yield desired-RHCP insertion losses of 1.17 and 0.57 dB and polarization extinction ratios of 9.55 and 7.47 dB at 9.672 and 10.636 GHz, respectively. Together, the controlled configuration analysis, modal interpretation, broadband-validated equivalent circuit, and free-space measurements establish a reproducible route to dual resonant, same-handedness circular-polarization-selective transmission in a compact connected bilayer unit cell. The same polarization-sensitive resonances provide a passive interface for receiver-front-end interference suppression and antenna-interrogated CFRP sensing, in which laminate-state and defect-induced loading are encoded as spectral and polarimetric perturbations^33^.

## Methods

### Electromagnetic simulation

Full-wave simulations were performed in CST Studio Suite using one periodic unit cell. Unit-cell boundaries were assigned to the x- and y-directed faces with ${P}_{x}$ = ${P}_{y}$ = 11 mm, representing an infinite two-dimensional array. The z-directed boundaries were set to open (add space), and Floquet ports were placed on the two z boundaries. Two fundamental modes were retained at each port. CST’s circular-polarization option defined the modes directly as LHCP and RHCP relative to the fixed +z axis; therefore, no transverse-electric (${{TE}}_{00}$)/transverse-magnetic (${{TM}}_{00}$)-to-circular transformation was applied. Normal incidence used θ = 0° and φ = 0°.

### Controlled design analysis and response extraction

Four configurations were simulated to isolate the two controlled geometrical factors: equal and unequal terminal arms, each combined with the simple and compound rear-layer transformations. Forward and backward co- and cross-polarized circular-basis coefficients were extracted from 8 to 12 GHz. The reported amplitude-based circular dichroism was calculated as CD = |T_++_| − |T_−−_|, and the two operating frequencies were selected as local CD maxima rather than by an arbitrary transmission threshold. Surface currents were evaluated at 9.672, 10.3 and 10.636 GHz to distinguish the lower predominantly symmetric mode, the mixed CD-null state and the upper predominantly antisymmetric mode.

### Equivalent-circuit formulation

The principal inductances and capacitances were estimated from the metallic path lengths, strip widths, front–back overlap area, substrate thickness and permittivity. These quantities were reduced to the even and odd modal values reported in the Results. The even and odd frequencies were fixed by the geometry-derived LC values; only modal damping, coupling, the slowly varying direct path and the weak fringe-associated coefficient were fitted by global least squares to forward-RHCP CST magnitude samples. Dielectric loss was estimated from the 3010 loss tangent and included within the total effective modal damping.

### Fabrication and free-space measurement

The 27 × 19 array was fabricated on 1.28 mm Rogers 3010 using standard printed-circuit processing. The sample was centered between two identical linearly polarized X-band horn antennas in an anechoic chamber and connected to a vector network analyzer. Forward co- and cross-polarized linear-basis coefficients were measured by selecting the transmitter and receiver orientations. Each measurement was normalized to the corresponding free-space transmission reference. The odd cell counts provide a physical center cell and symmetric truncation for alignment; the overall finite-array dimensions were selected as a practical electromagnetic, fabrication and measurement compromise rather than through a global finite-array optimization.

### Linear-to-circular basis transformation

The measured 2 × 2 linear-basis transmission matrix was converted to the circular basis using the Jones formalism and the time-harmonic and propagation conventions defined in the Results. The same convention was used for measurement and reciprocity analysis. Receiver-front-end transmitted powers were calculated from the complete co- and cross-polarized circular coefficients, so that the reported insertion loss, rejected-polarization attenuation and polarization extinction ratio include both modal components rather than only the co-polarized terms.

## Data Availability

The datasets generated and/or analyzed during the current study are not publicly available due to the large size and software-specific format of the raw full-wave simulation and experimental measurement files but are available from the corresponding author on reasonable request.

## Code availability

No custom code was developed or used in this study.
